# Growth Performance, Immune-Related and Antioxidant Genes Expression, and Gut Bacterial Abundance of Pacific White Leg Shrimp, *Litopenaeus vannamei,* Dietary Supplemented With Natural Astaxanthin

**DOI:** 10.3389/fphys.2022.874172

**Published:** 2022-06-23

**Authors:** Abdallah Tageldein Mansour, Mohamed Ashour, Eman M. Abbas, Ahmed Saud Alsaqufi, Mahmoud S. Kelany, Mohamed A. El-Sawy, Zaki Z. Sharawy

**Affiliations:** ^1^ Animal and Fish Production Department, College of Agricultural and Food Sciences, King Faisal University, Al Hofuf, Saudi Arabia; ^2^ Fish and Animal Production Department, Faculty of Agriculture (Saba Basha), Alexandria University, Alexandria, Egypt; ^3^ National Institute of Oceanography and Fisheries (NIOF), Cairo, Egypt

**Keywords:** astaxanthin, feed additives, immune response, antioxidant, bacterial abundance, marine shrimp

## Abstract

The current study examines the effect of dietary supplementation of ethanolic extract of *Arthrospira platensis* NIOF17/003, which is mainly natural astaxanthins (97.50%), on the growth performance, feed utilization, bacterial abundance, and immune-related and antioxidant gene expressions of the Pacific white leg shrimp, *Litopenaeus vannamei*. A total of 360 healthy *L. vannamei* postlarvae (0.19 ± 0.003 g) were divided into four groups (0, 2, 4, and 6 g natural astaxanthins/kg diet) each in three replicates, at an initial density of 30 PLs per tank (40 L capacity). The shrimp were fed the tested diets three times a day at a rate of 10% of their total body weight for 90 days. Diets supplemented with different astaxanthin levels significantly improved shrimp growth performance and feed conversion ratio compared to the control diet. No significant differences were observed in survival rates among all experimental groups. The immune-related genes (*prophenoloxidase*, *lysozyme*, *beta-glucan binding protein*, *transglutaminase*, and *crustin*) mRNA levels were significantly upregulated in groups fed with different concentrations of the natural astaxanthins in a dose-dependent manner. The *prophenoloxidase* gene is the highest immune-upregulated gene (14.71-fold change) in response to astaxanthin supplementation. The superoxide dismutase mRNA level was significantly increased with increasing dietary astaxanthin supplementation. In addition, increasing astaxanthin supplementation levels significantly reduced the count of heterotrophic bacteria and *Vibrio* spp*.* in the culture water and shrimp intestine. Overall, the current results concluded that diet supplementation with natural astaxanthin, extracted from *Arthrospira platensis*, enhanced the growth performance, immune response, and antioxidant status of *L. vannamei.*

## Introduction

Shrimp is currently one of the most important aquatic animals worldwide. Due to the increase in global demand, shrimp culture has developed intensively and has priority among the leading aquaculture sectors in many countries (Lukwambe et al., 2019; Abbas et al., 2020). Among all penaeid shrimp species, the Pacific white leg shrimp, *Litopenaeus vannamei,* is the widest species being extensively cultured (Sharawy et al., 2022), accounting for more than 70% of the global shrimp production (Li et al., 2018). However, there are numerous barriers to sustaining aquaculture development globally, including the feed industry, pollution, low survival rates, climatic changes, and poor water quality (Abo-Taleb et al., 2020; Alprol et al., 2021a; Alprol et al., 2021c; Hassan et al., 2021). To cope with the global increase of intensive shrimp farming, the shrimp feed industry has been developed using several strategies. Among these strategies, feed additive supplementation is one of the most important industries that has gained great importance for several shrimp species as growth promoters, immunity enhancers, and an alternative strategy for disease-fighting (Sharawy et al., 2020; Sharawy et al., 2022).

Recently, due to their high concentration of natural bioactive compounds, algal cells (microalgae and seaweeds) have attract great attention for utilization as feed additives, showing improvement in growth performance, feed utilization, and immunity stimulation of cultured animal species, besides improving the water quality (Ashour et al., 2020; Ashour et al., 2021; Mabrouk et al., 2021; Zaki et al., 2021; Mansour et al., 2022). Depending on the algal strain, algal cells contain protein with high essential amino acid content, lipids with high unsaturated fatty acid levels, and carbohydrates (polysaccharides, etc.), which are necessary compounds in shrimp feeding, growth, and metamorphosis (Liñán-Cabello et al., 2003; Wade et al., 2017). Among all the microalgae strains, *Arthrospira* (a filamentous cyanobacterium) is the richest microalgae species in many phytochemicals (Mansour et al., 2021). *Arthrospira platensis* contains high levels of essential amino acids, fatty acids, minerals, and pigments like phycocyanin and astaxanthin, which have important biological functions and serve in several industries (Madkour et al., 2012; El-Shouny et al., 2015; Osman et al., 2016). It could be used for the replacement of fishmeal in the diet of Pacific white shrimp, *Litopenaeus vannamei,* and the obtained results did not show any significant differences with partial or total replacements in the growth performance and feed utilization levels. In addition, the PUFAs was increased significantly with *A. platensis* treatments and the survival of *A. platensis* supplemented groups was significantly increased under hypoxia challenge (Pakravan et al., 2017). The hot-water extract of *A. platensis* improved the growth, genes expression, immune response, and resistance of *L. vannamei* against *Vibrio alginolyticus* (Lin et al., 2010; Tayag et al., 2010). Besides phycocyanin, astaxanthin is the main carotenoid that exists in *A. platensis* (Gouveia et al., 2003; An et al., 2017).

Astaxanthin, a xanthophyll carotenoid, is a fat-soluble red pigment that has more significant biological activities than other carotenoids (Lim et al., 2018). Astaxanthins are extensively used as feed additives in diets of juveniles and adults of several shrimp species. It could be resulting in improved growth performance, survival, feed utilization, immunity responses, digestive enzyme activities, body composition, reproductive performance, spermatophore, egg, and larval qualities, and overcoming the pigment deficiency of Pacific white leg shrimp, *L. vannamei* (Niu et al., 2009; Pei et al., 2009; Chuchird et al., 2015), kuruma shrimp, *Marsupenaeus japonicus* (Wang et al., 2019), *Penaeus monodon* (Chien et al., 2003), red cherry shrimp, *Neocaridina davidi* (Tomas et al., 2020). In addition, astaxanthin dietary supplementation has positive effects on growth, molting cycle, free radical scavenging capacity, and nitrite stress tolerance of *Penaeus japonicus* postlarvae (Petit et al., 1997) and *Pleoticus muelleri* (Díaz et al., 2014). Furthermore, natural astaxanthins derived from the green seaweed, *Enteromorpha intestinalis* were used as feed additives to increase astaxanthin content in shrimp, *Penaeus monodon*, muscles (Mondal et al., 2015). In the present study, astaxanthin was extracted from local strain of *A. platensis* NIOF17/003 as available, cheap, and sustainable source that can be used in a commercial scale as feed additives in shrimp diets. Recently, *A. platensis* was used as an efficient source for astaxanthin that can be increased *via* environment conditions manipulation (Moradi et al., 2021) or inducing mutation (An et al., 2017).

The immune systems of crustaceans depend on innate immunity, that is, mediated by cellular and humoral effectors, which recognize invading microorganisms and trigger various defense mechanisms to eliminate pathogens (Söderhäll and Cerenius, 1992; Mansour et al., 2021; Sharawy et al., 2022). Humoral effectors include the prophenoloxidase system (*ProPO*), hemolymph clotting mechanism, melanization, and antimicrobial immune response (Jiravanichpaisal et al., 2006; Cerenius et al., 2008). The high immune surveillance of invertebrates could be associated with high amounts of hemolymph carotenoids, which could regulate genes expression of several immune-related genes, in particular, the *ProPO* gene (Cornet et al., 2007; Babin et al., 2010), CuZn superoxide dismutase (*SOD*) gene (Han et al., 2016), and other immune genes. Dietary astaxanthin could partially alleviate oxidative stress *via* inducing relatively higher gene expression levels of antioxidant enzymes in *L. vannamei* (Zhang et al., 2013). Meanwhile, the commercially farmed crustaceans did not have the internal mechanism for the *de novo* synthesis of astaxanthin and did not have the access to obtain different carotenoid sources from the environment (Higuera-Ciapara et al., 2006; Seabra and Pedrosa, 2010). Accordingly, dietary supplementation with astaxanthin is a necessity in the formulated diet. Therefore, the current study aimed to investigate the effects of increasing supplementation levels of the acetonic extract of the *A. platensis* NIOF17/003, which mainly consists of natural astaxanthins, as a feed additive on the growth performance, feed utilization, immune-related genes expression, and bacterial abundance of Pacific white leg shrimp*.*

## Materials and Methods

### *Arthrospira platensis* NIOF17/003

Cyanobacterium, *Arthrospira platensis* NIOF17/003 (GenBank accession number: MW396472), isolated from the El-Khadra saline-alkaline Lake, Wadi El-Natrun, Egypt, was molecularly identified, and cultivated, as well as its potential applications in different fields, were determined as described previously (Alprol et al., 2021b; Hassan et al., 2021; Mabrouk et al., 2021; Zaki et al., 2021).

### Astaxanthins Extraction, Preparation, and Analysis

Natural astaxanthin, a carotenoid pigment, was extracted from the blue-green alga *A. platensis* NIOF17/003, according to Ju et al. (2009) with some modifications. Briefly, 1 kg of *A. platensis* fine dried powder was soaked in 100% acetone (10% w: v) and extracted three times on a rotary shaker for 72 h at 200 rpm in the dark at room temperature. The extracts were combined and filtered through filter paper (Whatman No. 1). Then the filtrates were concentrated using a rotary evaporator at 40°C under reduced pressure (Elshobary et al., 2020). The crude extract yield was weighed and calculated as a percentage of the initial sample weight. The yield of crude extract was stored at −20°C until further application. To determine the phytochemical profile of *A. platensis* crude extract, GC-Mass Spectrophotometry analysis was performed as previously described by Ashour et al. (2020). The unknown phytochemical compounds were identified based on comparing the obtained mass spectra with those available in the NIST library (National Institute of Standards and Technology, United States).

### Experimental Animals

Pacific white leg shrimp, *L. vannamei,* postlarvae (PLs) were brought from a private commercial shrimp hatchery located in Borg El-Arab, Alexandria City, Egypt, to the indoor facilities of the Suez Branch, National Institute of Oceanography and Fisheries (NIOF-Suez). Firstly, the PLs were acclimated for 2 weeks in fiberglass tanks (500 L) under the same controlled conditions of the feeding trial (26–28°C, 31–32 ppt, and continuous aeration). During the acclimated period, PLs were fed a commercial diet (Aller-Aqua, Giza Governorate, Egypt). The compliance with ethical standards in the experimental setup and fish handling was approved by the Research Committee of the NIOF, Egypt.

### Experimental Facilities and Design

According to a completely randomized design, the current feeding trial was performed in three replicates for each treatment. After 2 weeks of acclimation, 360 healthy PLs (0.19 ± 0.003 g) at an initial stocking density of 30 PLs per tank were handed out into 12 tanks (40 L capacity). During the experimental period (90 days), all PLs were fed three times a day (6:00, 12:00, and 18:00 h) at a rate of 10% of their total body weight. Every day before the first feeding, all tanks were siphoning to clean and remove the accumulated excreta and unconsumed feed. As a result of the siphoning process, 10% water was replaced daily with filtered, oxygenated seawater (Sharawy et al., 2020).

### Water Quality Parameters

During the feeding trial, water quality parameters were checked, twice a week, for alkalinity (mg/L), NH_3_ (mg/L), PO_4_ (mg/L), NO_3_ (mg/L), and NO_2_ (mg/L) as described by APHA (2005). In addition, to maintain the water quality values as recommended for shrimp, the temperature (°C, using a mercury thermometer suspended at a depth of 30 cm), pH (using a pH meter, Orion, United States), and salinity (ppt, using a refractometer, United States) were investigated daily (1.00 p.m.) (Boyd and Tucker, 2012).

### Diets Preparation

In the current feeding trial, four dietary supplementation treatments were performed for 90 days: D_1_, a control diet, that the shrimp was fed a commercial diet (Aller-Aqua, Giza Governorate, Egypt, as presented in Table 1). The other three diets (D_2_, D_3_, and D_4_) were fed diets supplemented with different levels of the natural astaxanthins (2, 4, and 6 g/kg diet, respectively), as a crude extract of *A. platensis* NIOF17/003. The addition of natural astaxanthins was performed as described by Mehrabi et al. (2012) with some modifications. The control diet was powdered and divided into four equal portions. The specific quantities of the natural astaxanthins (0, 2, 4, and 6 g/kg diet, respectively) were suspended well in 50 ml of corn oil and then sprayed well over the three powdered diets and mixed well. For the control diet (D1), the same volume of corn oil was sprayed without astaxanthins. Then, all four diets were re-pelletized in a pellet mill to obtain the proper diameter, dried at room temperature with forced air, and stored in plastic bags at 4°C until use.

**TABLE 1:** The proximate chemical analysis (% of dry matter) of experimented diets.

Diets^a^	Protein (%)	Fat (%)	Ash (%)	Fiber (%)
D_1_	40.59	11.31	9.19	3.51
D_2_	39.69	12.60	8.89	4.14
D_3_	39.88	12.28	9.21	3.48
D_4_	40.51	12.62	9.40	3.41

a Aller-Aqua, Giza Governorate, Egypt. D_1_, D_2_, D_3_, and D_4_ are the experimental diets that were supplemented with 0, 2, 4, and 6 g astaxanthins/kg diet, respectively, of the crude acetonic extract of *Arthrospira platensis* NIOF17/003.

### Measured Parameters

#### Growth Performance and Nutrient Utilization Indices

At the end of the experiment, the PLs weights were recorded to determine the final body weight (FBW, g). Moreover, to determine the growth performance of Pacific white leg shrimp, *L. vannamei*, the weight gain (WG), feed conversion ratio (FCR), survival (%), and specific growth rate (SGR) were calculated using the following formulas:

Weight gain, g= Final body weight (g)−Initial body weight (g) (1)

$$\text{Feed conversion ratio}=\frac{\text{Total consumed feed }}{\text{WG}}$$ (2)

$$\text{Survival},\text{\% }=\frac{\text{Final number of shrimp}}{\text{Initial number of shrimp}}\;\times 100$$ (3)

$$\text{Specific growth rate},\text{\%}/\text{day}=\frac{\text{Ln FBW }-\text{ Ln IBW}}{t}\;\times 100$$ (4)

where: Ln FBW and Ln IBW are the natural logarithm of final body weight (g) and initial body weight (g); while t is the time in days.

### Whole-Body Proximate Chemical Analysis

At the end of the experiment, to determine the whole-body proximate chemical composition, five shrimp from each replicate were selected randomly and homogenized by a blender, dried in an oven, ground, and stored at −20°C for subsequent analysis. Both biochemical analyses of shrimp and diet were applied as described by the standard methods of AOAC (2003). The shrimp, dry matter, crude protein, crude fat, and crude ash were determined, while for diets, crude protein, crude fat, crude ash, and fibre were determined, and the nitrogen-free extract was calculated.

### Bacterial Abundance Assessment

The bacterial abundance of water and shrimp intestines was performed according to APHA (2005). At the end of the experiment, three shrimp samples were chosen randomly from each replicate, and the intestines were aseptically extracted to estimate the bacterial count as described by Sharawy et al. (2020). The outwardly surface bacteria were removed by washing each gut three times with sterile distilled water. After that, they were washed in ethanol 96% and homogenized in a mortar separately. At the end of the experiment, samples of culture water (1 ml) and intestines (1 g) were taken from each treatment (three replicates) and supplied with sterile distilled water (9 ml). Later, make dilutions (1:10) and transferred 1–10 ml TSA (Trypticase soy agar) and TCBS (Thiosulphate-Citrate-Bile salts) agar plates and incubated at 37°C for TSA and 28°C TCBS (Sharawy et al., 2020). After 24 h, the colonies in each plate of the TSA and TCBS were counted, and the colonies of *Vibrio* spp*.* were confirmed using the 0129 test (Thermo Scientific™ Oxoid™ 0129 Discs) (Xie et al., 2020).

### Immune-Related Gene Expressions Analysis

Triplicate samples of the shrimp abdominal muscles from each replicate were directly excised with fully sterile dissecting tools under cold conditions. The samples were kept at −80°C until gene expression analysis. Total RNA was extracted from the samples using the TRIzol method (easy-RED, iNtRON Biotechnology) as directed by the manufacturer. The OD ratio at 260/280 nm of RNA purity was determined using a NanoDrop system (BioDrop), and the samples with the highest ratio (A260/A280 1.8) were used for cDNA synthesis (1 ng/µl) for each reaction. Total RNA was treated with DNase I (NEB, United States) as the template for the synthesis of first-strand cDNA using reverse transcriptase (RT-PCR beads, Enzynomics, Korea), and the reaction was carried out using PCR amplification (Applied Biosystems Veriti 96-Well Thermal Cycler, United States) under the manufacturer’s conditions. The following cDNA was used in the Real-Time PCR reaction (Bico, Thermo-Fisher): initial denaturation at 95°C for 15 min, 40 cycles with the following parameters (95°C, 10 s; 58–62°C, 20 s; and 72°C, 30 s). Unique and specific products were seen as a melting curve at the end of the last cycle when the temperature increased from (58–62–95°C) in increments of 0.5°C. The studied immune-related genes were prophenoloxidase (*proPO*), lysozyme (*Lys*), beta-glucan binding protein (*Bgp*), superoxide dismutase (*SOD*), transglutaminase (*TGase*), and crustin (*Crus*), and their primers were presented in Table 2. The housekeeping gene (β-actin) was used to measure gene expression or fold shift of the target genes (Yang et al., 2013). The values give n-fold difference relative to the calibrator (control) when the 2^ΔΔ^Ct method is applied in normalizing the critical threshold (Ct) quantities of target genes with quantities β-actin (Livak and Schmittgen, 2001).

**TABLE 2:** Primer sequences for real-time PCR used for gene expression analysis.

Gene	Primer sequence (5′-3′)	Accession no.	Size (bp)
*β-actin*	F: GCCCATCTACGAGGGATA	AF300705	121
	R: GGT​GGT​CGT​GAA​GGT​GTA​A		
Prophenoloxidase (*proPO*)	F: CGG​TGA​CAA​AGT​TCC​TCT​T	AY723296	122
	R: GCAGGTCGCCGTAGTAAG		
Lysozyme (*Lys*)	F: GGA​CTA​CGG​CAT​CTT​CCA​GA	AY170126	97
	R: ATC​GGA​CAT​CAG​ATC​GGA​AC		
Beta-glucan binding protein (*Bgp*)	F: ACG​AGA​ACG​GAC​AAG​AAG​TG	AY249858	137
	R: TTC​AGC​ATA​GAA​GCC​ATC​AGG		
Superoxide dismutase (*SOD*)	F: TCA​TGC​TTT​GCC​ACC​TCT​C	AY486424	143
	R: CCG​CTT​CAA​CCA​ACT​TCT​TC		
Transglutaminase (*TGase*)	F: TTC​ACA​AGC​CTG​ACA​TCA​CC	BE188522	99
	R: GCA​GCA​GTG​GGA​TAG​GGT​TA		
Crustin (*Crus*)	F: ACGAGGCAACCATGAAGG	AF430076	141
	R: AAC​CAC​CAC​CAA​CAC​CTA​C		

### Statistical Analysis

The experiment was performed in triplicates and the results of growth performances were presented as the means ± standard deviation (SD). The normality and homogeneity assumptions were confirmed before the statistical analysis of the data. Before analysis, all results in percentages were arc-sin transformed (Zar, 1984). Using the IBM SPSS Statistics software (IBM, v.23), statistical analysis was performed by the One-Way Analysis of Variance (ANOVA), followed by Duncan’s post-hoc test, at a significant *p* ≤ 0.05.

## Results

### Astaxanthin of *A. platensis* NIOF17/003

**FIGURE 1:**
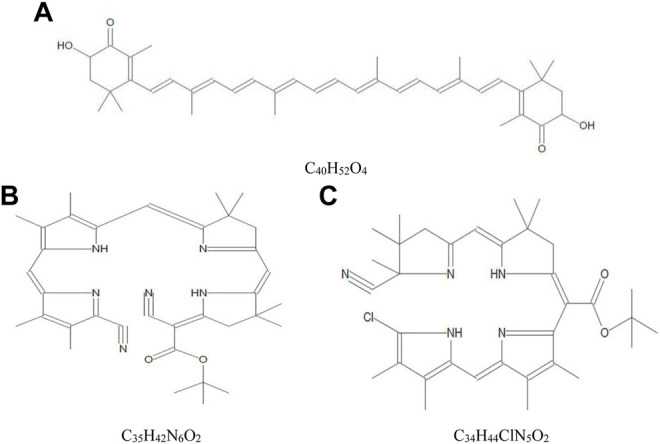
Mass spectra and chemical structure of the three phytochemical compounds identified in crude acetonic extract of *Arthrospira platensis* NIOF17/003. **(A)**: Astaxanthin; **(B)** Cyano 5-{5-[5-(5-cyano-3,4-dimethylpyrrol-2-ylidenemethyl)-3,4-dimethyl-1H-pyrrol-2-ylmethylene]-4,4-dimethyl-4,5-dihydro-3H-pyrro-2-ylmethylene}-4,4-dimethylpyrrolidin-2-ylene-acetic acid, tert.-butyl ester; and **(C)**: [5-(5-Chloro-3,4-dimethyl-1H-pyrrol-2-ylmethylene)-3,4-dimethyl-5H-pyrrol-2-yl]-[5-(5-cyano-4,4,5-trimethyl-4,5-dihydro-3H-pyrrol-2-ylmethylene)-4,4-dimethylpyrrolidin-2-ylene]-acetic acid, tert.-butyl ester.

The yield of crude extract of *A. platensis* NIOF17/003 was weighed and calculated as a percentage of the initial weight. The calculated final yield concentration was 27 g/kg (2.7%). The GC-MS analysis of the crude extract of *A. platensis* NIOF17/003 shows three main phytochemical compounds belonging to three retention times (Table 3). These different bioactive compounds were astaxanthin (C_40_H_52_O_4_, exact molecular weight: 596.38) with the highest peak area (97.50%) and the highest probability (21.40%). The peak area and probability of the other two bioactive compounds (C_35_H_42_N_6_O_2_ and C_34_H_44_ClN_5_O_2_) were 0.38%, and 0.65%, respectively, and the probability was 7.07%, and 6.60%, respectively (Table 3). The chemical structure of these three phytochemicals were identified using the NIST library as shown in Figure 1.

**TABLE 3:** Phytochemical profile investigated in crude extract of *A. platensis* NIOF17/003.

RT	PA%	Compound’s name	P%	CF	EMW
19.610	98.50	Astaxanthin	21.40	C_40_H_52_O_4_	596.38
19.739	0.85	Cyano 5-{5-[5-(5-cyano-3,4-dimethylpyrrol-2-ylidenemethyl)-3,4-dimethyl-1H-pyrrol-2-ylmethylene]-4,4-dimethyl-4,5-dihydro-3H-pyrro-2-ylmethylene}-4,4-dimethylpyrrolidin-2-ylene-acetic acid, tert.-butyl ester	7.07	C_35_H_42_N_6_O_2_	578.33
14.937	0.65	[5-(5-Chloro-3,4-dimethyl-1H-pyrrol-2-ylmethylene)-3,4-dimethyl-5H-pyrrol-2-yl]-[5-(5-cyano-4,4,5-trimethyl-4,5-dihydro-3H-pyrrol-2-ylmethylene)-4,4-dimethylpyrrolidin-2-ylene]-acetic acid, tert.-butyl ester	6.60	C_34_H_44_ClN_5_O_2_	589.31

RT, retention time; PA, peak area%; P%, Probability%; MF, molecular formula; and EMW, exact molecular weight.

### Water Quality Parameters

Table 4 shows the water quality parameters during the experiments. The results revealed that all recorded water quality conditions (°C, pH, salinity, alkalinity, NH_3_, PO_4_, NO_3_, and NO_2_) were in the recommended ranges for shrimp culture. No significant difference was observed among fish fed the control diet and the diets supplemented with different concentrations of astaxanthins.

**TABLE 4:** Mean values of water quality parameters during the feeding trial.

Water quality parameters	Experimental diets^a^			
	D_1_	D_2_	D_3_	D_4_
NH_3_ (mg L^−1^)	0.119 ± 0.001	0.116 ± 0.010	0.115 ± 0.015	0.103 ± 0.014
NO_2_ (mg L^−1^)	0.119 ± 0.016^a^	0.109 ± 0.001^ab^	0.108 ± 0.003^ab^	0.095 ± 0.015^b^
NO_3_ (mg L^−1^)	0.222 ± 0.028	0.219 ± 0.003	0.225 ± 0.007	0.214 ± 0.023
PO_4_ (mg L^−1^)	0.485 ± 0.009	0.495 ± 0.039	0.517 ± 0.022	0.494 ± 0.004
Alkalinity (mg L^−1^)	7.700 ± 0.625	7.725 ± 0.225	7.987 ± 0.137	8.337 ± 0.212
Temperature (°C)	26.84 ± 0.20^a^	26.55 ± 0.01^b^	26.57 ± 0.07^b^	26.65 ± 0.11^ab^
Salinity (ppt)	32.25 ± 0.09^b^	32.52 ± 0.02^a^	32.41 ± 0.07^ab^	32.52 ± 0.18^a^
pH	7.79 ± 0.02	7.77 ± 0.01	7.76 ± 0.01	7.76 ± 0.02

a D_1_, D_2_, D_3_, and D_4_ are the experimental diets that are supplemented with 0, 2, 4, and 6 g astaxanthins/kg diet, respectively, of the crude acetonic extract of *Arthrospira platensis* NIOF17/003.

Data are means ± SD and the *n* = 3. Different letters in the same row are significantly different (*p* < 0.05).

### Growth Performance and Nutrient Utilization Indices

**FIGURE 2:**
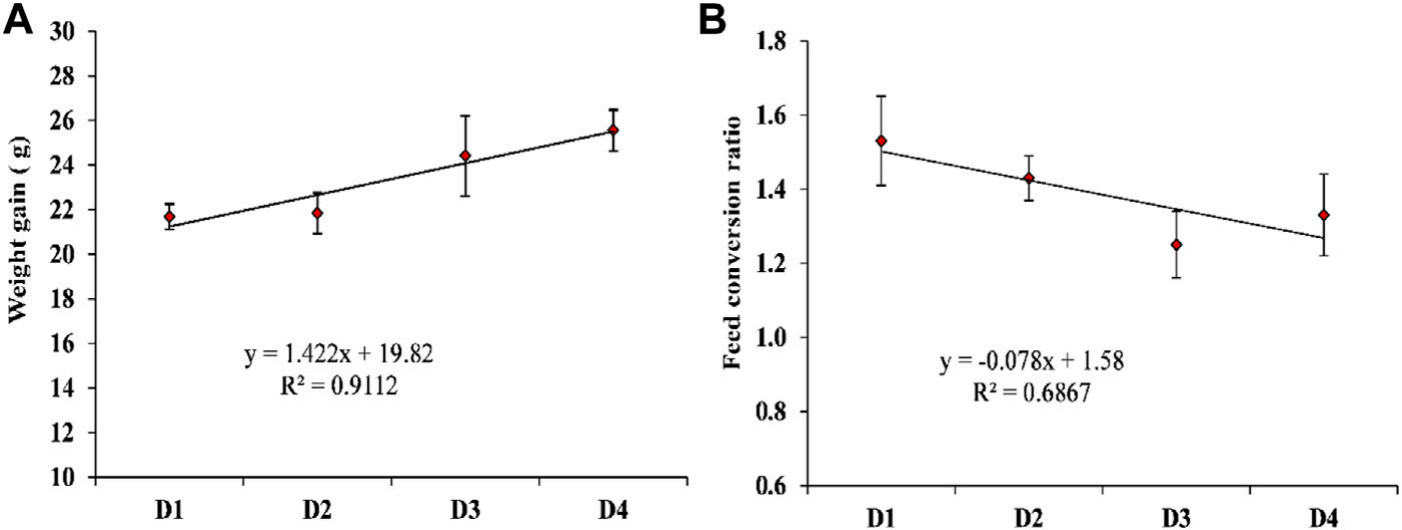
The appropriate regression models of the increasing inclusion levels of dietary astaxanthin supplementation for the **(A)** weight gain; **(B)** food conversion ratio of *L. vannamei.* D_1_, D_2_, D_3_, and D_4_ are the experimental diets that are supplemented with 0, 2, 4, and 6 g astaxanthin/kg diet, respectively, of the natural astaxanthins of the crude acetonic extract of *Arthrospira platensis* NIOF17/003. Data are means ± SD and the *n* = 3.

Table 5 shows the effect of dietary supplementation of astaxanthin on the growth performance and feed utilization of *L. vannamei* juveniles. Diets supplemented with different concentrations of astaxanthins (D_2_, D_3_, and D_4_) experienced a significant (*p* < 0.05) improvement of FW, WG, and FCR compared to the control diet. On the other hand, no significant differences (*p* < 0.05) were obtained in survival or SGR among the diets supplemented with astaxanthins (D_2_, D_3_, and D_4_) and the control. While, the response of shrimp, in terms of WG and FCR to increasing inclusion levels of dietary astaxanthin supplementation showed a linear regression pattern with a strong correlation for WG (*r* ^*2*^ = 0.9112) and a moderate correlation for FCR (*r* ^*2*^ = 0.6867), as presented in Figure 2.

**TABLE 5:** Growth performance and feed utilization of Pacific white leg shrimp, *Litopenaeus vannamei*, fed experimental diets for 90 days.

Growth indicators	Experimental diets^a^			
	D_1_	D_2_	D_3_	D_4_
IBW (g)	0.19 ± 0.003	0.19 ± 0.003	0.19 ± 0.003	0.19 ± 0.003
FBW (g)	21.87 ± 1.15^c^	22.03 ± 1.33^b^	24.61 ± 1.78^a^	25.75 ± 1.42^a^
WG (g)	21.68 ± 0.57^c^	21.84 ± 0.93^b^	24.42 ± 1.80^a^	25.56 ± 0.92^a^
Survival (%)	82.22 ± 3.85	76.67 ± 3.33	77.78 ± 1.92	74.44 ± 5.09
SGR (%/day)	5.27 ± 0.01	5.28 ± 0.038	5.40 ± 0.028	5.45 ± 0.06
FCR	1.53 ± 0.12^a^	1.43 ± 0.06^a^	1.25 ± 0.09^c^	1.33 ± 0.11^b^

a D_1_, D_2_, D_3_, and D_4_ are the experimental diets that are supplemented with 0, 2, 4, and 6 g astaxanthins/kg diet, respectively, of the crude acetonic extract of *Arthrospira platensis* NIOF17/003.

Data are means ± SD and the *n* = 3. Different letters in the same row are significantly different (*p* < 0.05).

IBW, initial body weight (g); FBW, final body weight (g); WG, weight gain (g); SGR, specific growth rate (%/day); and FCR, feed conversion ratio.

### Body Proximate Analysis

As presented in Table 6, there are significant differences (*p* > 0.05) that were reported in the whole-body chemical composition (dry matter, protein, fat, and ash content) of shrimp *L. vannamei*. The control group had the highest significant (*p* < 0.05) values of dry matter and crude protein content. While D_4_ showed the highest significant (*p* < 0.05) impacts on fat and ash content (Table 6).

**TABLE 6:** Proximate whole-body proximate analysis (% of wet weight) of Pacific white leg shrimp, *Litopenaeus vannamei,* fed experimental diets for 90 days.

Proximate composition indicators	Experimental diets^a^			
	D_1_	D_2_	D_3_	D_4_
Dry matter (%)	26.53 ± 0.10^a^	25.04 ± 0.04^b^	24.84 ± 0.12^c^	25.58 ± 0.07^b^
Protein (%)	23.12 ± 0.03^a^	21.77 ± 0.04^c^	21.56 ± 0.06^d^	22.19 ± 0.02^b^
Fat (%)	7.79 ± 0.01^d^	9.99 ± 0.02^c^	10.07 ± 0.04^b^	10.87 ± 0.03^a^
Ash (%)	1.60 ± 0.01^d^	1.99 ± 0.02^b^	1.92 ± 0.03^c^	2.08 ± 0.03^a^

a D_1_, D_2_, D_3_, and D_4_ are the experimental diets that are supplemented with 0, 2, 4, and 6 g astaxanthins/kg diet, respectively, of the crude acetonic extract of *Arthrospira platensis* NIOF17/003.

Data are means ± SD and the *n* = 3. Different letters in the same column are significantly different (*p* < 0.05).

### Bacterial Abundance Investigations

The effects of experimental diets supplemented with astaxanthin on both total heterotrophic bacteria (THB) and total *Vibrio* spp*.* count (TVC) in the water and intestine of *L. vannamei* are shown in Table 7. It was observed that the degradative heterotrophic bacteria were more abundant in the intestine than in water. Concerning pathogenic bacteria, the genus *Vibrio* spp*.* was chosen as an indicator for the pathogenicity of shrimp, and the count was lower than heterotrophic bacteria. However, when compared to the control, the THB count in the water and intestine decreased gradually as astaxanthin supplementation levels increased.

**TABLE 7:** Bacterial abundance in the culture water and intestine of Pacific white leg shrimp, *Litopenaeus vannamei,* fed experimental diets for 90 days.

Bacterial count	Experimental diets^a^			
	D_1_	D_2_	D_3_	D_4_
Water				
THB (cfu mL^1^ x10^4^)	95.90 ± 0.04^d^	41.90 ± 0.03^c^	26.50 ± 0.06^b^	11.90 ± 0.66^a^
TVC (cfu mL^1^ x10^3^)	11.40 ± 0.05^d^	6.80 ± 0.02^c^	4.50 ± 0.03^b^	0.5 ± 0.04^a^
Intestine				
THB (cfu g^1^ x10^4^)	399.90 ± 0.04^d^	249.90 ± 0.03^c^	119.90 ± 0.04^b^	30.00 ± 0.03^a^
TVC (cfu g^1^ x10^3^)	111.20 ± 0.4^d^	47.60 ± 0.40^c^	28.20 ± 0.60^b^	6.70 ± 0.30^a^

a D_1_, D_2_, D_3_, and D_4_ are the experimental diets that are supplemented with 0, 2, 4, and 6 g astaxanthins/kg diet, respectively, of the crude acetonic extract of *Arthrospira platensis* NIOF17/003.

Data are means ± SD and the *n* = 9. Different letters in the same row are significantly different (*p* < 0.05).

THB, total heterotrophic bacterial count; TVC, total *Vibrio* spp. count.

### Immune-Related Gene Expressions

**FIGURE 3:**
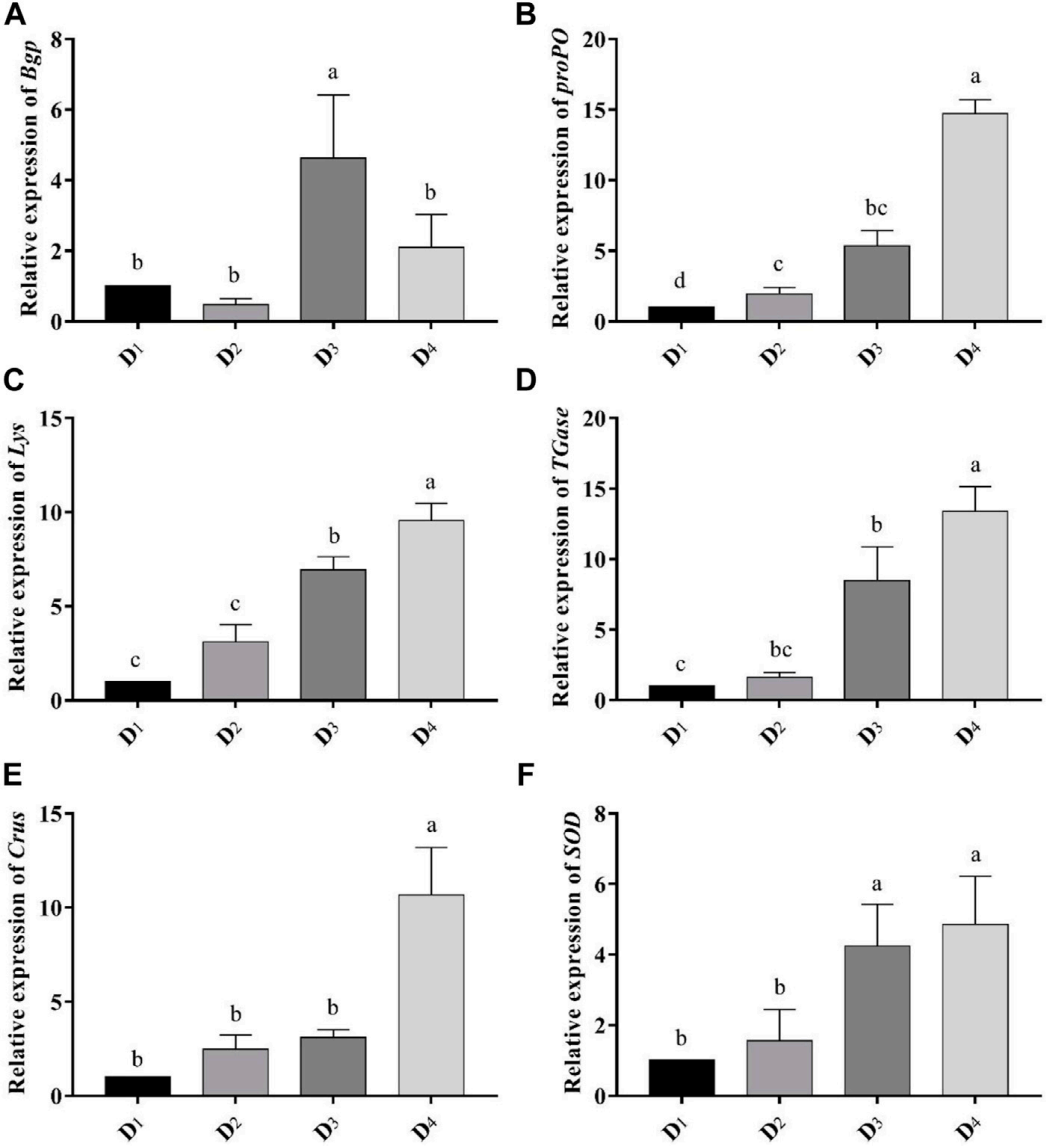
Analysis of immune-related gene-expressions [*Bgp* **(A)**, *proPO* **(B)**, *Lys* **(C)**, *TGase* **(D)**, *Crus* **(E)**, and *SOD* **(F)**] comparing to the expression of *β-actin* gene in the different inclusion levels of astaxanthin dietary supplementation. D_1_, D_2_, D_3_, and D_4_ are the experimental diets that are supplemented with 0, 2, 4, and 6 g astaxanthins/kg diet, respectively, of the crude acetonic extract of *Arthrospira platensis* NIOF17/003. The data are means ± SD and the *n* = 9. Different letters for the same gene indicated a significantly differences (*p* < 0.05).

Dietary inclusion of astaxanthin enhanced the expression of all studied genes: *Bgp*, *Lys*, *proPO*, *TGases*, *Crus*, and *SOD* in the muscle tissue of *L. vannamei* at the end of the feeding trial (Figure 3). The expression of the *Bgp* gene was significantly increased in the dietary supplemented treatment with astaxanthin at a level of 4 g/kg compared to the control group and D_2_ even though the expression in D_3_ was higher than D4. Generally, gene expression of *Lys*, *proPO*, *TGases*, *Crus*, and *SOD* was significantly upregulated (*p* < 0.05) with increasing the concentration level of natural astaxanthins in the diet compared to the control. The *proPO* gene expression was the most upregulated gene among the other genes, and the relative fold change was 14.71 compared to the control group.

## Discussion

Recently, with the growing development of aquaculture, feed additives demand is globally increased, resulting in an enormous space for the industrial application of natural astaxanthin (Lu et al., 2021). Generally, natural astaxanthins are extracted from different aquatic organisms such as shrimp (Scurria et al., 2020), soft coral (Metwally et al., 2020), fish (Yu and Liu, 2020), seaweeds (Teramukai et al., 2020), and microalgae (Molino et al., 2018). Among all aquatic organisms, microalgae have the highest ability to produce astaxanthin than any aquatic animal (Gallego et al., 2019). Microalgae are high productive microorganisms, carbon captures, and oxygen producers. Therefore, the reliance on the production of microalgae-based astaxanthin has positive environmental impacts in mitigating the effects of global warming and creating environmental benefits (Lee and Ding, 1994; Wu et al., 2017).

In the present study, the final yield of crude extract, mainly astaxanthin, of *A. platensis* was 2.7%. Compared to other microalgal species, *Haematococcus pluvialis* is considered the most widespread species that produces natural astaxanthin with a high final yield ranging from 3.8%–4% (Lee and Ding, 1994; Aflalo et al., 2007; Ranga Rao et al., 2010). Meanwhile, the final yield of natural astaxanthin in the present study exceeded that reported in several microalgae species, such as *Chlorella zofingiensis*, *Neochloris wimmeri*, *Chlorococcum* sp*.*, *Botryococcus braunii*, *Tetraselmis* sp*.*, and *Scenedesmus obliquus*, which produced 0.68% (Orosa et al., 2001), 0.60% (Orosa et al., 2000), 0.20% (Zhang et al., 1997), 0.01% (Grung and Metzger, 1994), 0.23% (Lim et al., 2018), and 0.30% (Qin et al., 2008), respectively. The yields of astaxanthin among different microalgae species vary due to several reasons, such as strain, extraction solvent, and extraction conditions. Accordingly, *A. platensis* NIOF17/003 could be considered as a source for producing natural astaxanthin.

Astaxanthin has been used as a dietary supplementation for aquatic animals (Niu et al., 2009; Lim et al., 2018). In the current study, the FW and WG, were significantly increased with the increase of astaxanthin supplementation levels compared to the control. In accordance with the present findings, Flores et al. (2007) reported that the inclusion of 80 mg/kg of synthetic astaxanthin enhanced the growth, survival, and molting frequency of *P. vannamei*. In addition, the inclusion of natural astaxanthin extracted from *H. pluvialis* significantly improves the growth performance, pigmentation, and antioxidant capacity of the white prawn *Exopalaemon carinicauda* (Zhang et al., 2021). Astaxanthins extracted from *H. pluvialis* and *A. platensis* strain Pacifica improved shrimp kuruma and *M. Japonicus* growth and hypoxia stress resistance (Chien and Shiau, 2005).

The maintenance of high immune surveillance is one of the crucial measures of successful aquatic cultured animals (Mansour et al., 2018; Mansour et al., 2020). In crustaceans, the innate immune system is the main animal defence to any pathogen. This defence is mediated by several kinds of cells, enzymes, and antimicrobial peptides (Huang et al., 2020). The present finding revealed an upregulation of the *Bgp* gene in shrimp fed 4 and 6 g astaxanthin/kg diets, especially with the 4 g/kg diet. The *Bgp* gene works as a vital factor for activation of the proPO system, coagulation progression, and expression of antimicrobial peptides after recognizing the microbial components (Goncalves et al., 2012). The *Bgp* gene showed a delayed upregulation in *L. vannamei* fed immunostimulant b-1, 3-glucan from *Schizophyllum commune* daily for a 1-week feeding trial (Wang et al., 2008). This contrast with the present findings could be due to the short treatment period with b-1,3-glucan than astaxanthin and the different mode of action of both treatments.

In addition, the *proPO* gene expression was significantly increased in shrimp fed 4 and 6 g/kg astaxanthin supplemented diets compared to the control group, and it was approximately 14-fold higher than the control, in the present study. Furthermore, the expression of *proPO* is the highest among all studied immune-related genes in the present study with increasing the concentration of astaxanthin (6 g/kg diet). Whereas, one of the most important components of the shrimp immune system is prophenoloxidase (Sritunyalucksana and Söderhäll, 2000).

Lysozyme can hydrolyze bacterial cell walls and operates as a non-specific innate defense molecule against bacterial infections. It has been demonstrated that it activates in penaeid shrimp in response to *Vibrio*, and its gene has been cloned and characterized in *L. vannamei* and *M. japonicas* (Hikima et al., 2003). In the present study, *Lys* gene expression in *L. vannamei* was increased gradually in the shrimps that fed with the three levels of astaxanthin supplemented diets. It was about 9-fold higher in the D_4_ treatment (6 g/kg diet) than in the control group. Kuruma shrimp fed a diet supplemented with astaxanthin experienced higher lysozyme activity, and total hemocyte count and improved the survival of shrimp against low salinity levels (Wang et al., 2019). Transglutaminase is recognized as an invertebrate defense mechanism. *TGase* gene silencing has previously been demonstrated to make shrimp susceptible to both bacterial and viral infections, indicating that *TGase* is an important component of the shrimp immune system (Fagutao et al., 2012). In this study, supplemented diets with natural astaxanthin influenced *TGase* gene expression and showed a significant upregulation in the fish fed 6 g/kg astaxanthin supplemented diet compared to the control group. In the same manner, *Crust* gene expression was improved with dietary supplementation of astaxanthin in a dose-dependent manner. Crustin is one of the antimicrobial peptides in penaeid shrimps hemolymph. After oral treatment for 7 days with peptidoglycan, a significant increase in crustin mRNA levels in *M. japonicas* was reported (Rattanachai et al., 2004). In the same line, Pacific white shrimp *L. vannamei*, fed diet supplemented with 80 mg astaxanthin/kg diet significantly improved serum phenoloxidase activity serum bacteriolytic activity, total haemocyte counts, and phagocytic activity (Wang et al., 2015). In addition, the antioxidant prosperities of astaxanthin (Figure 3F) could directly participate in the immune enhancement in *L. vannamei*. However, the immune-stimulating activity of astaxanthin in crustaceans still needs more investigation to better understand its mode of action.

Superoxide dismutase (SOD) is one of the main antioxidant enzymes responsible for scavenging reactive oxygen species and is considered a safeguarding mechanism inside the tissue that could be damaged by oxidation processes and phagocytosis (Chien et al., 2003). Because of its unique chemical structure, astaxanthin has the potential to has antioxidant effects, including free radicals scavenging and activating the expression and activities of several antioxidant enzymes (Eren et al., 2019; Yu et al., 2021). In our study, the expression of *SOD* was significantly upregulated in shrimp fed astaxanthins at levels of 4 and 6 g/kg compared to the control group. In line with the current findings, astaxanthin supplementation increased the expression levels of *Cyt-Mn SOD*, *CAT*, and *GPx* genes (Liu et al., 2018) and SOD activity in *L. vannamei* (Chuchird et al., 2015). Whereas, astaxanthins as a carotenoids reported to protect white blood cells from oxidative damage, enhancing cell-mediated, and humoral immune responses of vertebrates and invertebrates (Song et al., 2020). This refers to the antioxidant activity of carotenoids that may be involved in the immunomodulatory action by quenching singlet oxygen and free radicals (Cvetkovic et al., 2013).

Generally, the contents of the gut microbiota have a strong influence on the health of aquatic organisms (Sharawy et al., 2020), such as digestion, nutrient absorption, immunity responses, and biological antibiosis (Li et al., 2018). The intestinal bacteria respond quickly to changes in food consumption, diet composition, and ingredients (Ringø et al., 2016). In the current study, the counts of THB and TVC were significantly (*p* > 0.05) decreased with increasing the inclusion levels of astaxanthin, compared to the control diet. Whereas, the bacterial abundance of *Vibrio* spp. was decreased in all astaxanthin supplemented diets compared to the control. Chuchird et al. (2015) reported an increase in the survival, growth, and resistance to *V. parahaemolyticus* of *L. vannamei* fed an astaxanthin supplemented diet. Furthermore, shrimp-fed diets supplemented with astaxanthin had significantly lower total intestinal bacteria and *Vibrio* spp. counts (Chuchird et al., 2015). The mode of action by which astaxanthin affected the bacterial population still not clear and could need more investigation. All these indications are in line with our findings, which revealed that astaxanthin is a promising substance for controlling the pathogenic bacteria load during the whole culture process of shrimp. These results were attributed to the high biological activities of acetonic extract of *A. platensis* NIOF17/003, mainly astaxanthin, which make it a wonderful, sustainable, and eco-friendly feed additive for aquaculture applications.

## Conclusion

From the current findings, it could be concluded that the *Arthrospira platensis* NIOF17/003 strain (Accession GenBank number: MW396472) is a good source of astaxanthin with a high final yield of about 2.7%. Dietary supplementation with natural astaxanthin enhanced the growth, feed utilization, and chemical composition of Pacific white leg shrimp, *Litopenaeus vannamei*. In addition, astaxanthin proved a powerful immune stimulant, antioxidant, and antibacterial substance for *L. vanami*. More research is needed to determine the mechanism of astaxanthin’s immunostimulant effects in shrimp, including cytokines mediated humoral and cellular innate immunity.

## Data Availability

The data that support the findings of this study are available from the authors upon reasonable request.
